# Trifolium Flavonoids Overcome Gefitinib Resistance of Non-Small-Cell Lung Cancer Cell by Suppressing ERK and STAT3 Signaling Pathways

**DOI:** 10.1155/2020/2491304

**Published:** 2020-10-22

**Authors:** Zhiqiang Wu, Bin Xu, Zhiyi Yu, Qin He, Zhuyuan Hu, Shishi Zhou, Meiqin Chen, Liang Zhu

**Affiliations:** Jinhua Municipal Central Hospital, Affiliated Jinhua Hospital, Zhejiang University School of Medicine, China

## Abstract

Gefitinib is a tyrosine kinase inhibitor of EGFR (epidermal growth factor receptor) and represents the first-line treatment for EGFR mutation patients with NSCLC (non-small-cell lung cancer) therapeutics. However, NSCLC patients are inclined to develop acquired gefitinib drug resistance through nowadays, unarticulated mechanisms of chemoresistance. Here, we investigated the role of TF (Trifolium flavonoids) on sensitizing gefitinib resistance in NSCLC cells and revealed its potential mechanism of action. We demonstrated that TF exerted significantly potential chemosensitivity in gefitinib resistant NSCLC cells. MTT assay and cytological methods were used to analyze cell viability and apoptosis in NSCLC cell line PC-9R. Both TF and gefitinib suppressed PC-9R cell growth in a dose-dependent manner. Subtoxic concentrations of TF did significantly augment gefitinib-induced apoptosis in PC-9R cell line. The TF promoted chemosensitivity was major mediated by the PARP and caspases activation. Meanwhile, the TF promoted chemosensitivity also decreased the expression of Bcl-2 and Mcl-1. Finally, TF significantly reduced the phosphorylation levels of STAT3 and ERK. Altogether, the results of the present study indicated the potential mechanisms of chemosensitivity of TF in gefitinib-induced apoptosis of NSCLC by downregulating ERK and STAT3 signaling pathways and Bcl2 and Mcl-1 expression and a promising application of TF in therapy of NSCLC with gefitinib resistant.

## 1. Introduction

Globally, NSCLC (non-small-cell lung cancer) is the most common cause of cancer-related deaths, accounting for 85% of primary lung cancer cases, and approximately 65% are diagnosed at an advanced stage [1, 2]. It is well known that chemoresistance is the most important factor for therapeutic failure of NSCLC, and it also results in the drug resistance of NSCLC to gefitinib-based therapy, making NSCLC the most general reason of cancer-related mortality [3–5]. Therefore, the identification of new compound to sensitize NSCLC to chemotherapy especially gefitinib-based therapy is an urgent requirement.

Flavonoids are cytoprotection compounds that are current in dietary vegetables, plants, and fruits [6]. One of the most leading genera of the family *Fabaceae* is the genus *Trifolium*, which contains coumarins, terpenes, chalcones, isoflavonoids, and flavonoids [7–10]. Moreover, the previous study from both *in vitro* and *in vivo* has proved that flavonoids were distinguished antioxidant molecules, which could be a therapeutic tool for many cancer types including colorectal cancers, lung cancer, breast cancer, prostate cancer, and so on [11–16]. However, the effect of flavonoids on chemosensitivity of gefitinib resistance in NSCLC has been few reported.

At the present study, we found that TF suppressed PC-9R cell growth in a dose-dependent manner, the same as gefitinib. Furthermore, subtoxic concentration of TF did significantly augment gefitinib-induced apoptosis in PC-9R cells by cleaving the PARP and caspases and decreasing the expression of Bcl-2 and Mcl-1. Mechanistically, TF also significantly reduced the phosphorylation levels of STAT3 and ERK. Altogether, the results indicated the potential mechanisms on chemosensitivity of TF in gefitinib-induced apoptosis of NSCLC by downregulating ERK and STAT3 signaling pathways and the expression of antiapoptotic Bcl2 family members Bcl-2 and Mcl-1.

## 2. Materials and Methods

### 2.1. Cell Line and Culture Conditions

The human gefitinib-induced drug resistant NSCLC cell line PC-9R was cultured in DMEM (Dulbecco's modification of Eagle's medium Dulbecco, Gibco) supplemented with 10% FBS (Hyclone), 100 *μ*g/ml of streptomycin (Gibco) and 100 U/ml of penicillin (Gibco). The cells were cultured in a humidified incubator in an atmosphere of 5% (*v*/*v*) CO_2_ at 37°C.

### 2.2. Cell Viability Assay

TF and gefitinib were tested *in vitro* for cytotoxicity against gefitinib resistant NSCLC cell line PC-9R in 96-well plates by using the MTT assay. In brief, a number of 2500 cells/well were seeded into 96-well plates and cultured for 24 h. After that, the medium was replaced with variable concentrations of TF alone or combined with indicated dose of gefitinib in full growth medium. Cells were exposed to variable concentrations of TF alone or in combined with indicated doses of gefitinib for 24 h followed by the MTT assay to determine cell survival rates. Using a microplate reader, the absorbance of each sample was read at OD570 nm.

### 2.3. Apoptotic Assay by Flow Cytometry

Apoptosis was assessed by flow cytometry using externalization of FITC-labeled Annexin V and PI (propidium iodide). Briefly, after treatment with variable concentrations of TF alone or combined with indicated dose of gefitinib for 24 h, human gefitinib resistant NSCLC cell line PC-9R was collected and washed with ice-cold 1 × PBS before staining in 400 *μ*l solution containing Annexin V-FITC for 20 min in dark at room temperature and then staining PI for 5 min. Soon later, the fluorescent signal in the cells was tested by flow cytometry (FACSCanto, Becton Dickinson). Data analysis was showed by the software WinMDI 2.9.

### 2.4. Western Blotting

The human gefitinib resistant NSCLC cell line PC-9R was lysed in Laemmli lysis buffer (2.1 *μ*g/ml aprotinin, 0.5 *μ*g/ml leupeptin, 1 mM orthovanadate, 1% Triton X, 1 mM phenylmethylsulfonyl fluoride, and 4.9 mM MgCl_2_). The lysate was centrifuged at 14,000 rpm for 7 min and boiled for 10 min. Proteins were loaded in denaturing SDS–PAGE gels and transferred to PVDF membrane (Millipore). After blocking with 5% fat-free milk for 2 h at room temperature, the membranes were washed by 1 × PBS containing 0.1% Tween-20 (PBST) and incubated with primary antibodies (antibodies against PCNA (No.13110), PARP (No.9532), caspase3 (No.9662), caspase8 (No. 4927), caspase9 (No. 9504), Bcl-2 (No. 15071), Mcl-1 (No. 94296), STAT3 (No. 9139), p-STAT3 (No. 9145), AKT (No. 2920), p-AKT (No. 9611), ERK (No. 4695), and p-ERK (No.9101) were from Cell Signaling Technology and diluted at 1 : 1000) at 4°C for another 2 h at room temperature. After washing with 1 × PBST, the membrane was incubated with secondary antibodies conjugated with horseradish peroxidase for 1 h at room temperature. Finally, signals were visualized with enhanced chemiluminescence (ECL, Thermofisher). *β*-Actin was used as loading controls. Quantitative analysis expression of proteins was performed by the software ImageJ.

### 2.5. Statistical Analysis

All data are expressed as the means ± SD from three independent experiments at least. The GraphPad Prism 5.0 (GraphPad Software, Inc., La Jolla, CA, USA) was used to all statistical analyses. Statistical significance was determined by using two-sided Student's *t*-test, and ^∗^*P* < 0.05 was considered significant.

## 3. Results

### 3.1. TF Potentiated Chemosensitivity to Gefitinib in NSCLC Cell Line PC-9R

**Figure 1:**
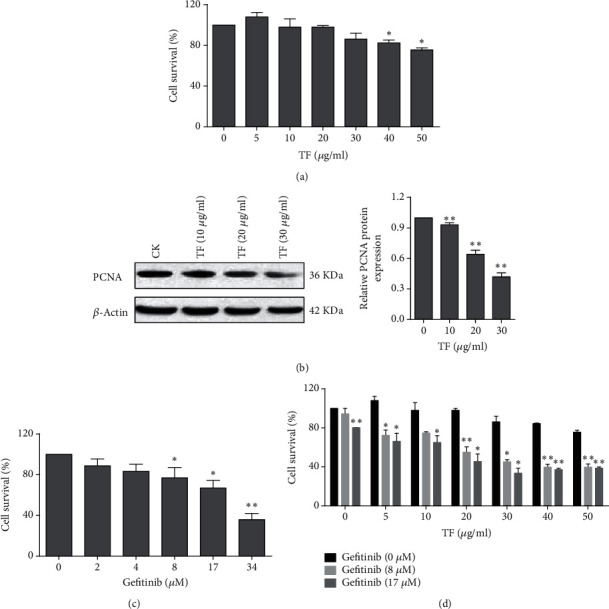
TF potentiated chemosensitivity to gefitinib in NSCLC cell line PC-9R. (a) TF inhibited human NSCLC cell growth *in vitro*. Cell survival was determined by MTT assay. (b) PC-9R cells were treated with indicated concentrations of TF for 24 h. Proteins were extracted and subjected to Western blotting assay to evaluate cleavage of caspases and PCNA (left panel); quantitative analysis expression of proteins presented (right panel). Data was presented by mean ± SD for three separate experiments. ^∗∗^*P* < 0.01. (c) Gefitinib inhibited human NSCLC cell growth *in vitro*. Cell survival was determined by MTT assay. (d) PC-9R cells were treated with vehicle, 8 *μ*M or 17 *μ*M gefitinib and then treated alone or combine with indicated concentration of TF for 24 h. Cell survival was determined by MTT assay. Each point represents the mean of the data of three independent experiments; bars, SD.^∗^*P* < 0.05; ^∗∗^*P* < 0.01.

To determine whether the TF could sensitize gefitinib-induced drug resistance or not, human gefitinib resistant NSCLC cell line PC-9R and gefitinib were obtained, and the cytotoxicity was evaluated by using MTT assay. The results revealed that TF could suppress PC-9R cell growth and the expression of cell proliferation marker PCNA by Western blot assay (Figures 1(a) and 1(b)). Our data also indicated that gefitinib inhibited the cell growth in a dose-dependent manner (Figure 1(c)). Additionally, we examined whether combined treatment of TF and gefitinib exerts enhanced lethality in PC-9R or not. As presented in Figure 1(d), after cotreatment with indicated concentrations of TF and gefitinib for 24 h and MTT assay was performed. Interestingly, combination of the subtoxic dose of TF and gefitinib dramatically inhibited the cell growth in PC-9R cells. Therefore, these results indicated that TF could effectively potentiate chemosensitivity of gefitinib-induced drug resistant NSCLC to gefitinib.

### 3.2. TF Potentiated Gefitinib-Induced Apoptosis in NSCLC PC-9R Cells

**Figure 2:**
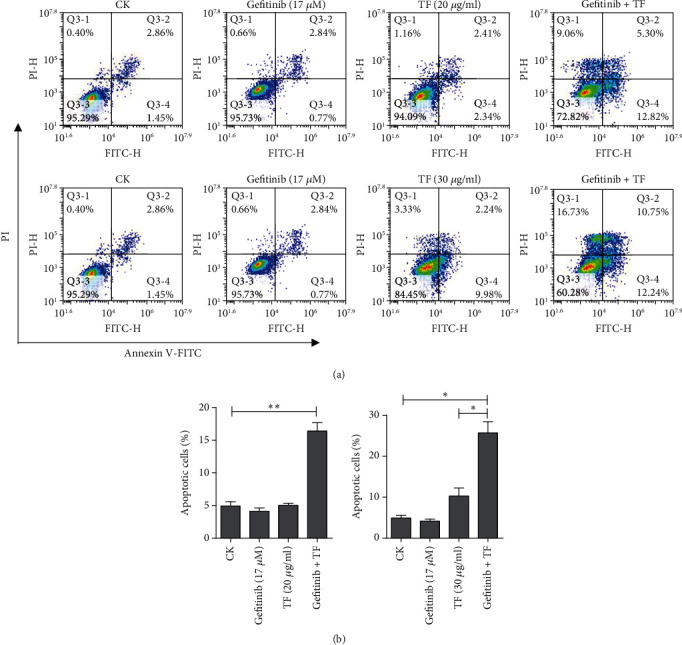
TF potentiated gefitinib-induced apoptosis in PC-9R cells. (a) PC-9R cells were treated with vehicle or 17 *μ*M gefitinib and then treated alone or combine with 20 *μ*g/ml or 30 *μ*g/ml TF for 24 h. The cells were then stained with Annexin V-FITC and PI and analyzed by flow cytometry. (b) Apoptosis was quantified and presented. Data were presented by mean ± SD for three separate experiments. ^∗^*P* < 0.05 vs. control, ^∗∗^*P* < 0.01 vs. control.

Next, we examined whether the sensitization effect of TF on gefitinib involved apoptosis induction; the PC-9R cells were subjected to flow cytometry after cells were treated with TF alone or in combination with gefitinib. As shown in Figure 2(a), TF significantly enhanced gefitinib-induced apoptosis in PC-9R, the percentage of Annexin V positive cells probably increased from 3.61% (17 *μ*M gefitinib alone) and 4.75% (20 *μ*g/ml TF alone) or 12.22% (30 *μ*g/ml TF alone) to 18.12% (17 *μ*M gefitinib combined with 20 *μ*g/ml TF) and 22.99% (17 *μ*M gefitinib combined with 30 *μ*g/ml TF) shown in Figure 2(b). These results suggested that TF significantly potentiated the cytotoxicity of gefitinib to PC-9R via inducing apoptosis.

### 3.3. Cleavage of PARP and Caspases Were Involved in the Synergistic Effect of TF on Gefitinib in PC-9R Cells

**Figure 3:**
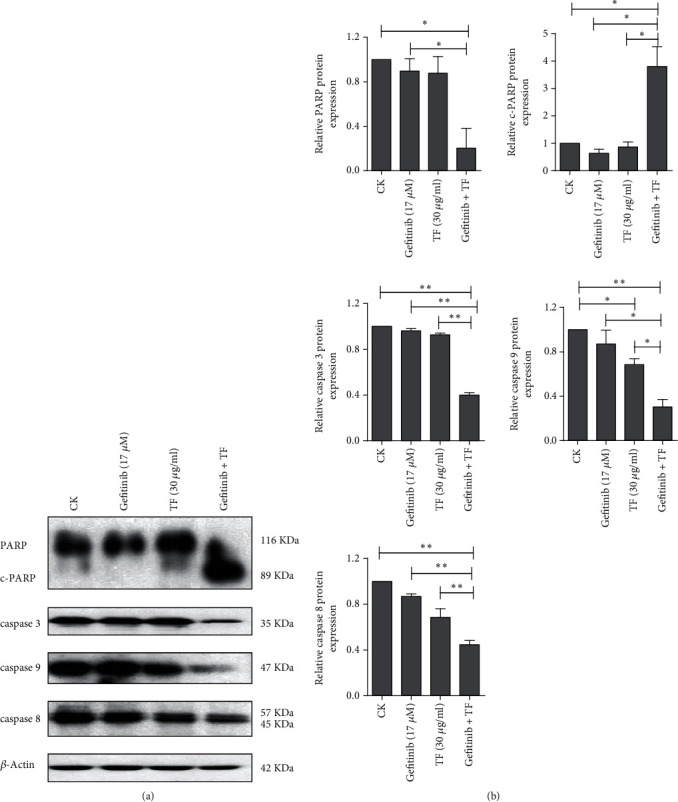
The combination of TF and gefitinib synergistically activated the cleavage of PARP and caspases. (a) PC-9R cells were treated with 17 *μ*M gefitinib alone or combine with 30 *μ*g/ml TF for 24 h. Proteins were extracted and subjected to Western blotting assay to evaluate cleavage of caspases and PARP. (b) Quantitative analysis expression of proteins in (a). Data was presented by mean ± SD for three separate experiments. ^∗^*P* < 0.05; ^∗∗^*P* < 0.01.

Cleavage of PARP and caspases is the hallmarks of activation of the apoptosis pathway [17]. As shown in Figure 3(a), cotreatment with 17 *μ*M gefitinib and 30 *μ*g/ml TF in PC-9R cells significantly increased the cleavage of PARP, caspase 3, caspase 9, and caspase 8. And quantitative analysis of proteins was shown in the histogram calculated by ImageJ software (Figure 3(b)). These observations indicated that cleavage of PARP and caspases was involved in the synergistic effect of TF on gefitinib in PC-9R.

### 3.4. Bcl-2 and Mcl-1 Were the Target by which TF Can Potentiate NSCLC to Gefitinib

**Figure 4:**
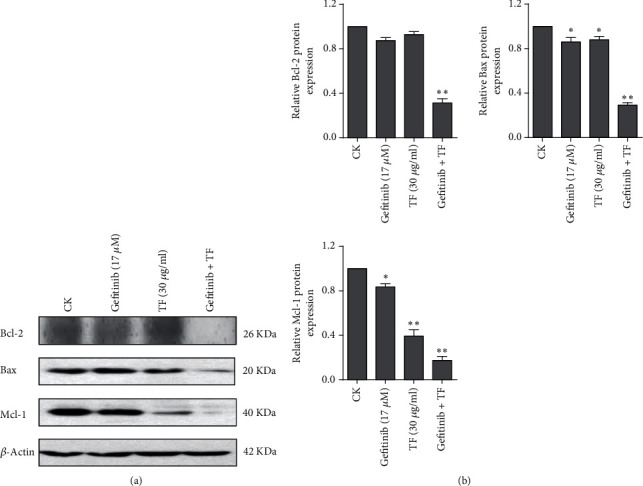
The combination of TF and gefitinib synergistically inhibited the expression of Bcl-2 and Mcl-1. (a) PC-9R cells were treated with 17 *μ*M gefitinib alone or combine with 30 *μ*g/ml TF for 24 h. Proteins were extracted and subjected to Western blotting assay to evaluate Bcl-2 and Mcl-1. (b) Quantitative analysis expression of proteins in (a). Data was presented by mean ± SD for three separate experiments. ^∗^*P* < 0.05; ^∗∗^*P* < 0.01.

Bcl-2 family is an important participant in apoptosis pathways [18]. As shown in Figure 4(a), cotreatment with 17 *μ*M gefitinib and 30 *μ*g/ml TF in PC-9R cell significantly decreased Bcl-2 and Mcl-1 protein levels. And quantitative analysis of proteins was shown in the histogram calculated by ImageJ software (Figure 4(b)). These observations indicated that Bcl-2 and Mcl-1 were key participant in the synergistic effect of TF on gefitinib in PC-9R.

### 3.5. TF-Induced Apoptosis in NSCLC via Inhibition of P-STAT3 and P-ERK Signaling Pathways

**Figure 5:**
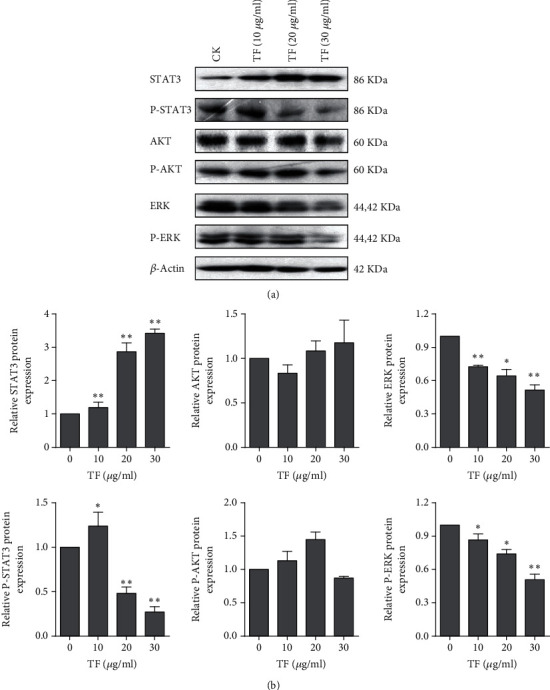
TF inhibited phosphorylation of STAT3 and ERK in PC-9R cells. (a) PC-9R cells were treated with indicated doses of TF for 24 h. Proteins were extracted and subjected to Western blotting assay to evaluate phosphorylation of STAT3, AKT, and ERK. (b) Quantitative analysis expression of proteins in (a). Data was presented by mean ± SD for three separate experiments. ^∗^*P* < 0.05; ^∗∗^*P* < 0.01.

As we know, the STAT3, AKT, and ERK signaling pathways are important for the balance between cell survival and apoptosis [19–21]. To determine whether STAT3, AKT, and ERK signaling pathways are involved in TF-induced apoptosis, we checked phosphorylated of STAT3, AKT, and ERK by Western blot assay (Figure 5(a)). TF treatment significantly decreased the level of phosphorylation of STAT3 and ERK but did not change the phosphorylation of AKT in PC-9R cells. As shown in Figure 5(b), the quantitative analysis of protein was shown in the histogram calculated by ImageJ software. These observations indicated that STAT3 and ERK signaling pathways might be a potential target by which TF can potentiate NSCLC to gefitinib.

## 4. Discussion

Gefitinib is one of the standard treatments for NSCLC with advanced EGFR mutation, which is typically administered to patients harboring L858R point mutations or exon 19 deletions and presents sophisticated efficacy on antitumor [4, 22, 23]. However, in almost all cases, patients whose treated with gefitinib would experience disease recurrence after 12 to 24 months due to acquired resistance [24]. Therefore, there is in sore need of new strategies to treat NSCLC patients with gefitinib resistance.

One such strategy is to use another compound to sensitize NSCLC to gefitinib-induced apoptosis. Flavonoids are a wide class of polyphenols, which can exert effects in cancer chemoprevention because of their capability to interfere with signaling cascades responsible for carcinogenesis and metastasis that has been connected with multiple cell-regulatory activities in cancer cells [25–27]. Flavonoid compounds have been tested against cancer patients under varieties of clinical trials. For instance, dietary flavonoid intake along with esophageal and gastric cancer incidence and survival in the United States of America was inspected [28]. Therefore, the synergistic effects of TF with gefitinib and the underlying mechanism were investigated in the present study.

Firstly, our data declared that TF inhibited NSCLC cell growth in a dose-dependent manner. Further, we illustrated the subtoxic level of TF sensitized NSCLC cell PC-9R to different concentrations of gefitinib by MTT assay. Next, we illuminated the synergistic effect of TF on gefitinib in apoptosis induction in PC-9R cell line, which were sent to flow cytometry analysis after treated with TF alone or in combination with gefitinib. In addition, we checked the cleavage of PARP, caspase 3, caspase 8, and caspase 9 and the decreased expression of Bcl-2 and Mcl-1 in the PC-9R cells cotreated with TF and gefitinib. Finally, we investigated the STAT3, AKT, and ERK signaling pathways under TF treatment. Our study demonstrated that TF could significantly descend the phosphorylation level of STAT3 and ERK but did not alter the phosphorylation of AKT in PC-9R cells.

## 5. Conclusions

Although others target genes of TF may also participate in inducing apoptosis via STAT3 and ERK pathways, the present data showed that TF could potentiates chemosensitivity in human NSCLC by suppressing Bcl-2 and Mcl-1 atleast partially, which served a prominent function as an activator in apoptosis by cleavage of PARP and caspases. Therefore, the results of the present study verified that the TF with gefitinib therapies may turn out to be effective new strategies for treatment with gefitinib-induced drug resistance in NSCLC.

## Data Availability

The data used to support the findings of this study are included within the article.
